# Sprint Speed Measurement in a National Rugby Union Academy: An Equivalence Study of Three Measurement Systems, Age, and Playing Position

**DOI:** 10.1002/ejsc.70154

**Published:** 2026-03-20

**Authors:** Andrew R. Wright, Matthew Weston, Fraser A. Menzies, Carlos Ramirez‐Lopez, Andy J. Boyd, Steve Barrett, Anthony P. Turner

**Affiliations:** ^1^ Institute for Sport Physical Education and Health Sciences University of Edinburgh Edinburgh UK; ^2^ Institute of Sport Manchester Metropolitan University Manchester UK; ^3^ High Performance Department Scottish Rugby Union Murrayfield Stadium Edinburgh UK; ^4^ Carnegie Applied Rugby Research (CARR) Centre Leeds Beckett University Leeds UK; ^5^ Playermaker London UK

**Keywords:** assessment, momentum, team sports, velocity

## Abstract

This study profiled the sprint speed of young male rugby union players in a national pathway academy, comparing different technologies for determining maximal velocity (*V*_max_), while also examining age‐group and positional effects. Data were collected from 140 players using a 40 m sprint during a single testing day. The data included anthropometric measures (age, body mass, and height), split times (10 m, 20 m, 30 m, and 40 m), maximum momentum, *V*_max_, and time to Vmax (tVmax), captured across three measurement systems: timing gates, local positioning systems (LPS), and foot‐mounted inertial measurement units (IMU). Effects were interpreted using equivalence testing. Here, regions of practical equivalence were informed by practitioner opinion of the acceptable amount of measurement error when measuring maximal speed and target change values for sprint times. Measurement comparisons showed practical equivalence only for Vmax between timing gates and LPS, suggesting they could be used interchangeably. All age‐group and positional differences were not practically important with 10‐m metre sprint times being practically equivalent between backs and forwards. Our findings emphasise the importance of measurement choice during speed profiling to inform speed and momentum development for young rugby players.

## Research Highlights

- Measurement systems showed practical equivalence only between Timing Gates and LPS for determining Vmax with a lack of practical equivalence for Vmax and tVmax when measured with IMU.
- In 40 m sprints, national academy U18 rugby union players were faster than U16 across all sprint splits with backs also faster than forwards. Results were not practically equivalent between age groups and positions, yet our novel analysis also highlights that the differences may not be practically important.

## 1 Introduction

Strength, power, aerobic fitness, and speed are key physical qualities required in rugby union to meet sport‐specific demands (Posthumus et al. 2020). Speed, in particular, is an important determinant of successful match‐play across a season in elite teams (Smart et al. 2014). Positional differences have been extensively characterised, with forwards exhibiting greater strength and power compared to backs who display greater speed (Cahill et al. 2013; Posthumus et al. 2020). Backs also cover higher relative and absolute distances, consisting of sprints and high intensity runs (Yamamoto et al. 2017), whereas forwards sustain more collisions via tackles and breakdowns (Cousins et al. 2023). However, both positional groups average ∼20 sprints per match (Campbell et al. 2018), highlighting the importance of speed and its development in rugby union across all levels of competition (Read et al. 2017).

Given the importance of sprint speed, various technologies have been used to profile speed in rugby union. Timing gates are commonly used to measure 10–40 m sprint performance (Turiman et al. 2022), based on practicality and affordability (Zabaloy et al. 2024), enabling the assessment of key variables; velocity, acceleration, time to peak velocity and momentum (Darrall‐Jones et al. 2015). Alternatively, global (GPS ‐ outdoor) and local (LPS ‐ indoor) positioning systems are often used in rugby union environments, due to high availability with devices commonly used to monitor loads during match‐play and training (Bridgeman and Gill 2021). Furthermore, GPS and LPS have the capacity to allow for more detailed speed profiling by capturing instantaneous velocity (Roe et al. 2017), providing higher data resolution than averaged split‐times when using timing gates. Importantly for practitioners, systems have provided valid and reliable data when used for profiling athletes from different team sports (Hoppe et al. 2018; Pino‐Ortega et al. 2022; Roe et al. 2017). However, recent evaluations highlight GPS‐derived speed metrics are sensitive to methodological factors (e.g., sampling frequency, processing methods; Zabaloy et al. 2024).

The use of inertial measurement units (IMUs) containing accelerometers, gyroscopes, and magnetometers, with high sampling frequencies (1000 Hz), has recently emerged as an alternative method for speed profiling. Current perspectives show practitioners see potential in their utility to track gait characteristics (Shushan et al. 2023), such as contact time, flight time and stride length, which are not provided using other tools such as timing gates, GPS, and LPS. Locomotor characteristics have demonstrated good inter‐unit reliability (Waldron et al. 2021; Myhill et al. 2023). However, this technology has shown underestimations of total distance at higher speeds in soccer players, compared to a GPS system (Polar Team Pro, Polar, Finland; Sandæl & Dalen, 2003). Differences between systems may be because of the positioning of the device (Macadam et al. 2019) and the measurements used to calculate them. Practitioners' knowledge on the interchangeability of systems is important as practitioners begin to use foot‐mounted IMUs more. For example, to understand differences in technical actions during training and between competition levels (Marris et al. 2022; Myhill et al. 2023). However, existing research on IMU advancements is limited (Macadam et al. 2019) and focused on football. Given this limited evidence base and measurement inconsistencies, direct comparison of systems within a rugby union population will inform practitioners' decision making on speed measurement and interpretation.

Equivalence testing is more appropriate than traditional tests of statistical difference when the research goal is methods comparison (Dixon et al. 2018). Further, statistically significant differences do not necessarily indicate practical relevance (Rappelt et al. 2025). Results of an equivalence test can be determined by a visual inspection of the confidence interval of the difference (Lakens et al. 2018) with effects declared as *practically equivalent* (90% confidence interval lies completely within the equivalence region), not *practically equivalent* (confidence interval overlaps the equivalence region), or *practically important* (confidence interval completely outside the equivalence region) (Lakens et al. 2018; Ganju and Rom 2017). This approach requires careful determination of the region of practical equivalence. While standardisation (e.g., 0.2*between‐subject standard deviations) reflects a common approach, simple effect sizes are recommended as they are independent of variance, easy to compute, and scale the original units which maximises practical context (Baguley 2009). Further, standardisation underestimates values of interest when compared to other approaches such as evidence syntheses, minimal detectable change scores, and opinion seeking (Kyprianou et al. 2019; Datson et al. 2022). Opinion seeking represents a valid method for determining practically meaningful effects (Cook et al. 2018) and offers a critical counterpoint to interpretation solely on statistical grounds (Datson et al. 2022).

Alongside advances in speed monitoring technologies, practitioners require sprint profiles to guide player monitoring and development. Speed development typically peaks in players' early 20s, highlighting its importance in both long‐term athlete development and talent identification (Barr et al. 2014). For example, players with faster 20 m sprint times were 1.4x more likely to be selected into a rugby union academy (Dimundo, Cole, Blagrove, McAuley, et al. 2021), with maximal speed distinguishing high‐performing from low‐performing rugby union players (Dimundo, Cole, Blagrove, Till, et al. 2021). Furthermore, from academy to professional level, speed demonstrates variations across age groups and playing positions (Owen et al. 2023; Zabaloy et al. 2021), with higher body mass negatively associated with maximum velocity (Barr et al. 2014; Darrall‐Jones et al. 2016). While body mass development improves momentum, itself a determinant of winning collisions (Cunningham et al. 2016), it must be balanced alongside speed development as players progress. Speed profiling therefore remains critical in evaluating players and monitoring training‐induced changes through player development pathways, particularly as junior players grow, mature and develop toward higher levels of competition.

Using equivalence testing anchored by practitioner opinion, we compared three different methods for determining maximal velocity while also profiling the sprint speed of young, male rugby union players engaged in a national pathway academy. Such analysis provides insight into the practical application of different technologies when measuring and interpreting sprint performance in rugby union players, while informing player monitoring and development within a national academy.

## 2 Materials and Methods

### 2.1 Research Design

Consistent with previous rugby sprint research (Barr et al. 2014; Cross et al. 2015; Darrall‐Jones et al. 2016) the study employed a cross‐sectional design, utilising a single testing day at the start of the 2024/25 rugby season to capture data across different groups of a national rugby union pathway academy. Testing was conducted in the morning, due to logistical constraints, while following guidelines to minimise the sequence effect and provide inter‐test rest (McGuigan 2016). The institutional ethics committee approved the study, and administrative consent was provided by the national rugby union for provision of fully anonymised datasets, under the conditions of individual player contracts.

### 2.2 Participants

Our sample consisted of 140 male players engaged in the Scottish national rugby union pathway academy and classified as Tier 3 on the McKay classification framework (McKay et al. 2021). The players represented two age categories and two positions (Under 16, U16, *n* = 101 [backs *n* = 47, forwards *n* = 54]; Under 18, U18, *n* = 39 [backs *n* = 20, forwards *n* = 19]). Our sample size is justified by resource constraints in that we only had access to a small but select sample of top athletes (Lakens 2022). Inclusion criteria ensured athletes were injury‐free and available for testing with no reported absences on the day of testing.

### 2.3 Procedures

Following assessment of body mass (Seca Alpha Model 875, Seca, Birmingham, UK) and stature (Seca 213 stadiometer, Seca, Birmingham, UK), players performed a 10 min standardised warm‐up consisting of dynamic exercises progressing in intensity and specificity for sprint performance. Linear sprint speed was then assessed over 40 m on an indoor, naturally‐ventilated 4G synthetic pitch to control environmental factors, such as weather (65% relative humidity, 1009 mbar pressure and 15°C) and surface (James et al. 2005). Players completed three maximal sprints on a 2 m‐wide 40 m‐long sprint lane that was marked using a tape measure, with a 0.5 m start distance and 10 m splits (i.e., 10 m, 20 m, 30 m, 40 m). Players started in a two‐point stance on the 0.5 m start distance that reduces the likelihood of false signals from limbs breaking the infrared beam (Haugen and Buchheit 2016). Like protocols employed by Darrall‐Jones et al. (2016), players started in their own time, sprinting as fast as possible through the 40 m gate, slowing only after passing the final gate. Players performed a walk back recovery with ∼5 min of rest and received split times between efforts.

Sprint performance was assessed simultaneously using three different approaches:

1. Timing gates (Witty Timing System, Microgate, Milan, Italy), spaced 2 m apart at 50 cm stand height, positioned at 0 m, 10 m, 20 m, 30 and 40 m, recorded split timings. Timing gate derived 10 m splits have demonstrated valid and reliable results (ICC 0.98, TEE 1.7%, CV 1.3%) in team sport athletes (Haugen et al. 2020; Roe et al. 2017).
2. LPS units (Vector S7 Units, Catapult Sports, Melbourne, Australia) measured instantaneous velocities at a frequency of 10 Hz. The LPS units sat between the scapulae within a specially designed vest and housed in manufacturer silicone straps. According to published guidelines (Hoppe et al. 2018; Rico‐González et al. 2020) devices were activated prior to the warm‐up, 15 min before testing with signals transmitted to in situ anchors (ClearSky, Catapult Sports, Melbourne, Australia) positioned around the pitch. LPS data was captured live using the manufacturer's software (OpenField version 3.10.5, Catapult Sports, Melbourne, Australia). LPS units have been shown to be valid and reliable (TEE 1.9% and CV 1.98%–2.12%) for velocity measures (Pino‐Ortega et al. 2022; Roe et al. 2017).
3. IMU devices (Playermaker, Tel Aviv, Israel) also recorded instantaneous velocity at 1000 Hz. IMUs were fitted to both feet over the player's boots below the lateral malleolus, following manufacturer's guidelines. Software for these devices used the manufacturer's app, version 3.50.1 and online dashboard v3.45.1.1. In line with Waldron et al. (2021) IMUs were activated before the warm‐up, 15 min prior to testing and required no calibration by the user prior to collection. Validity and reliability of these IMU devices (ICC values of 0.84–1.0) has previously been reported (Myhill et al. 2023; Waldron et al. 2021).

### 2.4 Data Processing

Data were processed using a consistent method to allow for potential future analysis (Varley et al. 2017). The best of the three sprint efforts, defined as the quickest timing gate trial was used for the age group (i.e., U16, U18) and positional analysis (i.e., backs, forwards), in line with previous research (Darrall‐Jones et al. 2016; Jones et al. 2018). To ensure consistency, the same sprint attempt and corresponding metrics were utilised for the technology comparison (timing gates, LPS, IMU). Velocity was determined by dividing distance between splits by change in time, and momentum was calculated by multiplying body mass and split velocity (kg·m·s^−1^). LPS data required identification of sprint start, defined as an exponential increase in velocity above 0.2 m.s^−1^ (Hoppe et al. 2018). Maximum velocity (*V*_max_) was subsequently determined to be the maximum instantaneous velocity achieved after sprint start (Hoppe et al. 2018; Roe et al. 2017) with time to Vmax (tVmax) as the time between sprint start and *V*_max_. IMU data were processed using the manufacturer's artificial intelligence software, which applied Kalman filtering to derive velocity (Vmax and tVmax) from gait metrics.

These outputs from each technology were exported from respective proprietary software and combined in Excel (Excel, Microsoft, Washington) by sprint trial for subsequent data inspection and statistical analysis. Visual inspection reviewed and cleaned data, accounting for four missing body mass values, with consistency checks to ensure data integrity.

### 2.5 Statistical Analysis

Descriptive data are presented as raw data points for each participant, group means ± standard deviations (SD) and boxplots. Missing data were negligible as from a total of 1820 potential study data points, only 12 were missing (height, *n* = 4; body mass, *n* = 4; maximum momentum, *n* = 4) representing 0.7% of the total dataset (Tierney and Cook 2023).

Using the observed data (Jakobsen et al. 2017), stepwise processes determined best model fit for the methods comparison and for explaining age‐related or positional variability in sprint times and maximum momentum. Model fit for the methods comparison was improved with the inclusion of a random player intercept but not the inclusion of a random slope, suggesting that the data structure required a mixed model approach but that the relationship between player and outcome did not vary across the different methods. The final model included method (IMU, LPS, Timing Gates) as a fixed effect and a random intercept for player with effects interpreted via equivalence testing. The *V*_max_ region of practical equivalence was 0.2 m.s^−1^ as this represents the median minimum acceptable amount of *V*_max_ measurement error reported by 50 practitioners with a median of 8 (IQR 5, 12) years' experience working worldwide in elite soccer (Kyprianou et al. 2019). In the absence of an opinion‐based anchor for t*V*_max_, we used the target change value for 40 m (0.258 s) as the t*V*_max_ equivalence region.

A model with age group and position as non‐interacted fixed effects best explained variability in sprint times and maximum momentum. Here, regions of practical equivalence were inferred from the change values in sprint times deemed practically important by 30 practitioners with a median of 3 (IQR 2, 6) years' experience working worldwide in elite female soccer (Datson et al. 2022). There is a strong linear relationship of sprint split times up to 40 m; regressing sprint distance (10 m, 20 m, 30 m, 40 m) with the time data of Haugen et al. (2020) returns a R^2^ of 0.986. Therefore, we regressed the Datson et al. (2022) sprint distances onto the times (0.09 s for 5 m, 0.21 s for 30 m) to predict 10 m, 20 m, and 40 m sprint times. From the resulting regression equation (*y* = 0.0048x + 0.066) values of 0.114 s (10 m), 0.162 s (20 m), and 0.258 s (40 m) represented our regions of practical equivalence. In the absence of practitioner opinion of a meaningful change in maximum momentum, interpretation is based on the location of the 95% confidence interval against a null difference. All model assumptions were checked using the *performance* package (Lüdecke et al. 2021), revealing evidence of heteroscedasticity for some split times; therefore, robust standard errors, clustered by age and position, were derived via the *sandwich* package where necessary (Zeileis et al. 2020). For all models, estimated marginal means and pairwise comparisons were obtained using the *emmeans* package (Lenth 2024). Visualisations and analyses were performed in R (version 4.1.2, R Foundation for Statistical Computing).

## 3 Results

**FIGURE 1:**
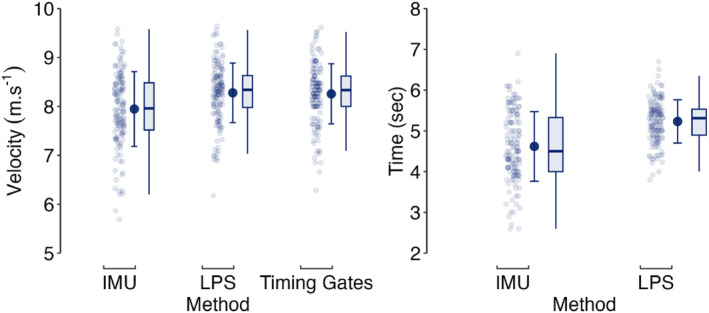
Descriptive (individual data points [smaller circles], mean ± SD [larger circle and error bar], and boxplots) for maximum velocity (left panel) and time to maximum velocity (right panel) data as measured by different methods.

**FIGURE 2:**
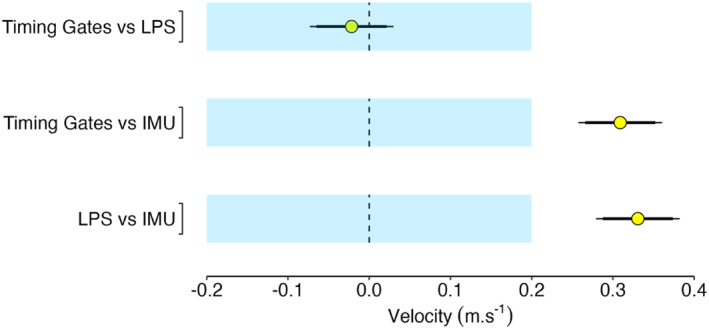
Mean between‐method differences (yellow dot) with 95% (thin line) and 90% (thick line) confidence intervals for maximum velocity. The region of practical equivalence is represented by the light blue shaded area.

**FIGURE 3:**
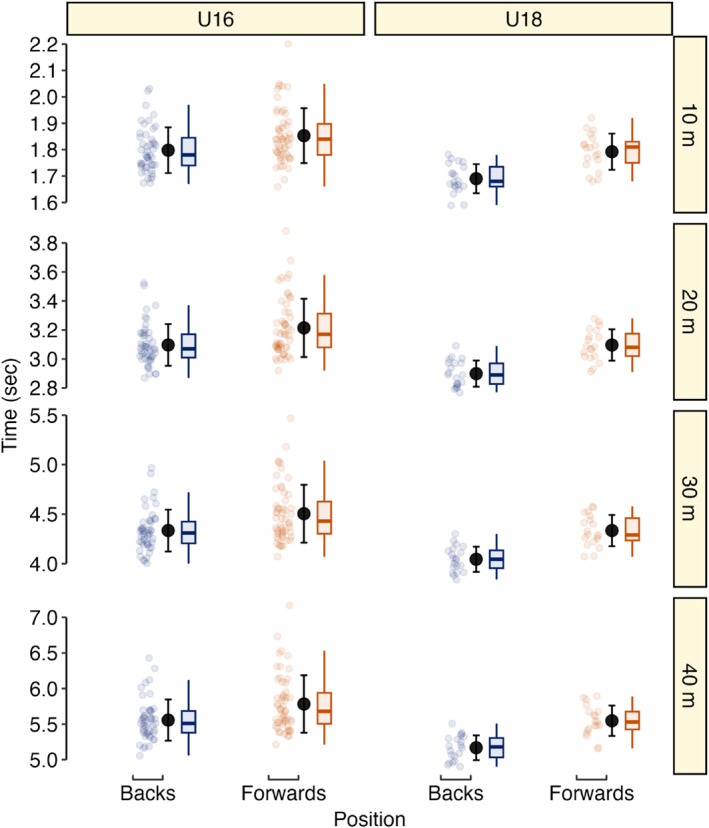
Descriptive (individual data points [smaller blue and red circles], mean ± SD [larger circle and error bar], and boxplots) 10 m, 20 m, 30 m, 40 m sprint times for U16 and U18 players.

**FIGURE 4:**
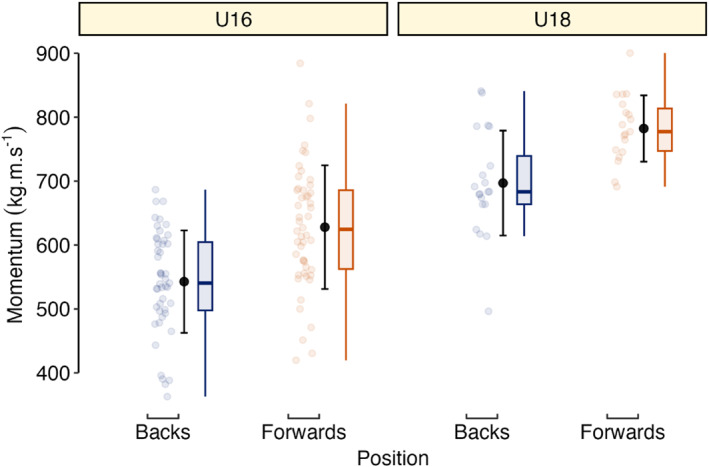
Descriptive (individual data points [smaller blue and red circles], mean ± SD [larger circle and error bar], and boxplots) maximum momentum data for U16 and U18 players.

**FIGURE 5:**
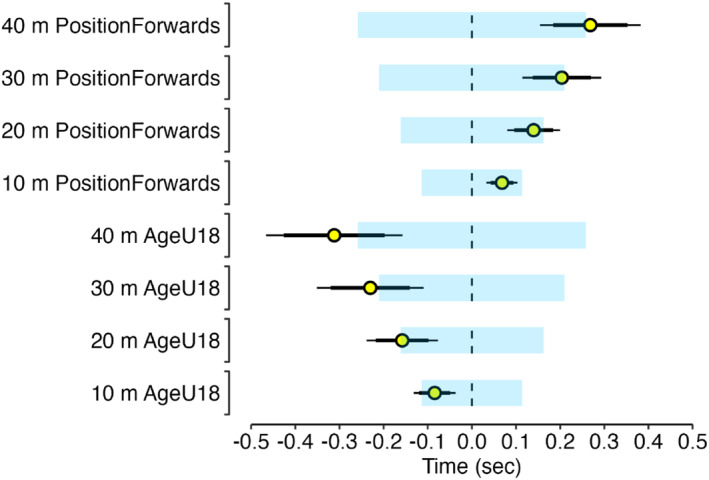
Age group (U18 v U16) and positional (backs v forwards) fixed effect differences (yellow circle) with 95% (thin line) and 90% (thick line) confidence intervals for 10 m, 20 m, 30 m, and 40 m sprint times. The region of practical equivalence for each sprint distance is represented by the light blue shaded area.

Descriptive player anthropometric, Vmax and tVmax data are presented in Table 1 and Figure 1. Timing Gates and LPS were practically equivalent for measuring Vmax (Figure 2) with IMU Vmax differences being practically important. For tVmax measurement, a 90% confidence interval for the difference ranging from 0.48 to 0.74 s indicates a practically important difference in these methods. Player sprint and maximum momentum data are presented in Table 1, Figure 3, and Figure 4. Ten‐metre sprint times were practically equivalent between backs and forwards, with all age‐group and positional differences declared as not practically important (Figure 5). Maximum momentum (Figure 4) was higher for U18 compared to U16 (154.3 kg·m·s^−1^; 95% confidence interval 122.9, 185.6 kg·m·s^−1^) and for forwards compared to backs (85.2 kg·m·s^−1^; 95% confidence interval 56.9, 113.6 kg·m·s^−1^).

**TABLE 1:** Anthropometric descriptive data, presented as means ± SD.

	Age groups		Positions	
	U16	U18	Forwards	Backs
Age (years)	14.6 ± 0.6	16.2 ± 0.7	15.0 ± 0.9	15.0 ± 1.0
Mass (kg)	72.7 ± 13.1	85.7 ± 11.2	83.5 ± 13.6	69.1 ± 9.8
Height (cm)	177.8 ± 7.7	181.1 ± 6.3	181.5 ± 7.1	175.7 ± 6.8

Abbreviations: U16 = under 16; U18 = under 18; cm = centimetres; kg = kilogrammes.

## 4 Discussion

This study profiled the 40 m sprint performance of male rugby union players engaged in a national pathway academy. It is the first one to employ regions of practical equivalence to evaluate measurement system agreement alongside exploring the practical importance of observed differences between age groups and positions. Firstly, method comparisons showed practical equivalence only between Timing Gates and LPS for determining *V*_max_. Secondly, our novel analysis highlights that the age‐group and positional differences were not practically important. Thirdly, when factoring‐in changes in body mass, despite being slower, forwards' maximum momentum was greater than backs, and U18 had higher momentum than U16.

Given the importance of Vmax in rugby union (Darrall‐Jones et al. 2016; Duthie et al. 2006), understanding differences between measurement systems is useful. The different values observed across measurement systems have practical significance for longitudinal tracking of player development, to ensure that observed changes in velocity metrics are due to true change in physical attributes rather than measurement discrepancy between various systems. The current study demonstrated practical equivalence between Timing Gates and LPS for measuring Vmax, suggesting the systems could be used interchangeably. Although, as far as possible, the same measurement method should be employed to establish a consistent system (Roe et al. 2017). In time‐constrained testing, each system presents distinct advantages and limitations. Timing gates offer immediate feedback, only require an initial set‐up, and are also more affordable for organisations outside performance sport; whereas LPS provide continuous measurement and additional metrics but require individual unit assignment which may represent an implementation barrier if working with large groups and a limited number of units. However, Vmax derived from IMU data were not practically equivalent to values from LPS and timing gates, with IMU under‐reporting Vmax compared to timing gates and LPS. Myhill et al. (2023) also reported IMU lacked agreement with a criterion (i.e., motion capture technology) at high speeds during team‐sport activities.

Practical equivalence was also not achieved between LPS and IMU for the measurement of tVmax, with longer times for LPS than IMU. These discrepancies may stem from differences in software algorithms or methodological procedures across measurement systems, particularly with sprint‐start thresholds that may capture the initiation of sprints differently. LPS used manual identification of sprint start by the lead researcher using a previously identified threshold (Hoppe et al. 2018), while IMU used artificial intelligence with set parameters. Additionally, IMU had a higher recording frequency compared to LPS, therefore potentially allowing for more precise identification of sprint start, although this would then be more likely to result in longer tVmax in IMU and this was not observed. Therefore, further investigation is recommended for tVmax using the same methodological procedure for measurement systems and higher sampling frequencies of LPS to identify sprint start.

We also recommend research into the technical factors influencing this tVmax, such as stride length and frequency which are both captured by IMU and would provide a comprehensive understanding of sprint profiles across individual rugby union athletes. Such insights may help to identify individual strengths and weaknesses between age groups and positions, offering a basis for training programmes tailored to positional demands and long‐term athletic development. Indeed, meta‐analyses emphasise the importance of sprint technique in terms of successful methods for improving sprint performance in team sports including rugby union (Nicholson et al. 2021). Foot‐mounted IMUs could therefore prove highly valuable, providing access to easily collected technique‐related data for example stride length, contact time, and flight time. These metrics offer practitioners a practical tool to monitor and refine sprint mechanics in youth rugby players, complementing traditional speed profiling and targeted training interventions. Longitudinal studies could further explore how technique‐related sprint mechanics evolve beyond the U18 level, providing insight into development alongside maturation and training exposure.

Sprint performance was not practically equivalent between age groups as U18 recorded faster sprint times than U16, which aligns with previous data from a different rugby academy (Darrall‐Jones et al. 2016). While our age effects failed to reach practical importance, we believe our findings underscore the importance of incorporating speed development in junior rugby players. Targeted speed training at this stage may help players maximise progress during a critical window for adaptation to prepare for higher levels of competition and before peak development in players' early 20s (Barr et al. 2014). Therefore, it is imperative to quantify and target speed development during this phase to meet the increased demands of higher competition levels (Duthie et al. 2006; Read et al. 2017). Sprint speed was also not practically equivalent between playing position as backs recorded faster times than forwards. Similarly, these effects failed to reach practical importance, but the data reinforce the positional demands for speed in rugby union (Barr et al. 2015; Posthumus et al. 2020), as backs experience greater high‐speed running demands during matches than forwards (Yamamoto et al. 2017).

While rugby is multifaceted, measuring sprint speed holds promise for coaches in talent identification (Dimundo, Cole, Blagrove, McAuley, et al. 2021; Dimundo, Cole, Blagrove, Till, et al. 2021). Although recent studies investigated sub‐positional differences (Owen et al. 2023), future research could further refine talent identification and development practices by applying regions of practical equivalence. However, evidence from youth rugby league populations suggest longitudinal development of absolute sprint speed is limited (Till et al. 2015; Wild et al. 2026). This indicates observation of sprint performance, alongside related measures (e.g., sprint momentum) may provide better insight into the physical maturation of pathway players. In the current study, older players demonstrated higher maximum momentum, explained by both faster velocity and greater body mass, consistent with Darrall‐Jones et al. (2015). This supports the notion that increased body mass alongside velocity development contributes to greater momentum for U18s. This finding is relevant in rugby as players progress to senior level, where greater momentum may be advantageous as competition levels increase (Read et al. 2017). This reinforces the importance of progressive, position‐specific training in youth rugby union settings (Barr et al. 2014; Owen et al. 2023); despite slower sprint speeds, forwards displayed higher maximum momentum than backs, identifying momentum as a key positional differentiator (Darrall‐Jones et al. 2016). However, although higher momentum is related to collision success, an increased body mass can negatively affect speed (Owen et al. 2020), highlighting a trade‐off across positions (Darrall‐Jones et al. 2016). Therefore, maximising momentum through increased body mass may be more critical for forwards and ball‐carrying backs (Barr et al. 2014). Future research should move beyond cross‐sectional designs, utilising longitudinal approaches with larger cohorts to observe the speed‐momentum relationship within individuals over multiple seasons, as well as across age groups and positions, as recently explored in rugby league (Wild et al. 2026). This would provide insight into the pathway's effectiveness in fostering practically important differences as an athlete progresses in age and training experience.

Our study is not without its limitations. Firstly, the application of our findings to the broader population of young rugby players across other organisations is limited by the sample being from a single national academy, as well as the sample being limited to only two age groups. Uneven balance between the age groups also poses a slight challenge. Although conversely, this sample reflects certain regional academies from the national pathway and reflects the natural reduction in player pool as athletes progress through age groups and levels toward to senior international levels. Secondly, the cross‐sectional nature of our research design limits a causal explanation of our data. There are likely within age‐group growth and maturation contributing mechanisms with regards to speed development, although with less impact than other sports as rugby players typically exhibit as early‐maturers (e.g., Howard et al. 2016). Thirdly, while our analysis considered general positional groups, demands differ between specific positions even within the groupings of forwards and backs (Reardon et al. 2015; Yamamoto et al. 2020). Finally, despite our athletes sharing similarities, that is, team‐based athletes ≥ Tier 3, with those of Datson et al. (2022), future work is needed to determine acceptable target change values for our population.

## 5 Practical Applications

In terms of practical guidance on selecting and implementing measurement systems to evaluate speed profiles across positions and age groups in young male rugby union pathway players, the practical equivalence observed between timing gates and LPS for measuring Vmax suggests that either system can be used in performance monitoring over 40 m, provided consistent protocols are followed (Roe et al. 2017). However, caution is warranted when using IMU for Vmax, as devices did not achieve practical equivalence with timing gates and LPS. Future studies should continue to explore discrepancies observed in tVmax, and investigate additional metrics from IMU, to further enhance the accuracy and reliability of sprint performance assessments. Although age‐group differences did not reach practical importance, profiling may still inform training. Particularly, U16 players may benefit from targeted speed training to minimise emerging disparities with U18 players during a key developmental period. Similarly, while sprint speeds were not practically equivalent between positions, momentum may offer a more position‐specific measurement to support positional development.

## 6 Conclusion

This study provides insight into sprint performance profiling within a national rugby union pathway, highlighting the importance of measurement and analysis approaches. Practical equivalence between timing gates and LPS for Vmax suggests interchangeable use, while IMU systems require further refinement for velocity measurement. Exploration of age and position differences did not reach the threshold for practical importance but emphasised differences in momentum (U18 > U16; forwards > backs). This analysis provides coaches and practitioners with a knowledge base to better evaluate and monitor player development, with the potential to inform training programmes. Future studies should look to explore longitudinal changes and investigate the sources of variability in tVmax across measurement systems, including use of IMUs to characterise changes in running stride metrics. Expanding sample sizes to include additional age groups while ensuring uniformity across groups would enhance the understanding and application of findings in national rugby union pathway players.

## Funding

The authors have nothing to report.

## Ethics Statement

The research received ethical approval from the Moray House School of Education & Sport Ethics Committee, University of Edinburgh.

## Consent

The administrative consent was provided by the national rugby union for provision of fully anonymised datasets, under the conditions of individual player contracts.

## Conflicts of Interest

The author SB is an employee of the company which manufactures the foot‐mounted IMU device in the current study. However, throughout the data collection and write up periods, to remove any bias, this author was not involved in any statistical analysis or data interpretation conducted within the current study.

## Data Availability

The authors have nothing to report.
